# The mitochondrial genome of the threatened tideland snail *Pirenella pupiformis* (Mollusca: Caenogastropoda: Potamididae) determined by shotgun sequencing

**DOI:** 10.1080/23802359.2022.2060143

**Published:** 2022-04-08

**Authors:** Shintaro Kato, Hajime Itoh, Hiroaki Fukumori, Nobuyoshi Nakajima, Gen Kanaya, Shigeaki Kojima

**Affiliations:** a Atmosphere and Ocean Research Institute (AORI), The University of Tokyo, Kashiwa, Chiba, Japan; b National Institute for Environmental Studies (NIES), Tsukuba, Ibaraki, Japan

**Keywords:** Cerithioidea, Gastropoda, next-generation sequencing, phylogenetic tree, *Pirenella pupiformis*

## Abstract

The nearly complete mitochondrial genome of the threatened tideland snail *Pirenella pupiformis* (Mollusca: Cerithioidea: Potamididae) was determined by shotgun next-generation sequencing. The mitogenome is comprised of 13 protein-coding genes (PCGs), two ribosomal RNA (12S and 16S) genes, and 22 transfer RNA genes (tRNAs). This gene order is consistent with the previously published mitochondrial genomes of other species belonging to the family Potamididae. The family Potamididae including *P. pupiformis* was recovered as a monophyletic group in the superfamily Cerithioidea.

Members of the potamidid gastropod genus *Pirenella* (Caenogastropoda: Cerithioidea) are found on tidal flats and mangroves of the western Pacific and Indian Oceans, and eastern Mediterranean Sea (Reid and Ozawa 2016). *Pirenella pupiformis* Ozawa & Reid, 2016 is a tideland species distributed from Japan, South Korea, China to Vietnam regions and recently described from Mie, Japan (Reid and Ozawa 2016). This species is currently included in the Red List of Japan (Ministry of the Environment, Japan 2020) and those of 17 Japanese prefectures (Search System of Japanese Red Data; http://www.jpnrdb.com/), and its genetic diversity and gene flow have been investigated for monitoring the conservation of local populations (Kojima et al. 2006, 2008; Kamimura et al. 2010). Regarding the previous mitogenomic study, approximately half of the mitogenome sequence of this species (7,750 bp, as *Cerithidea djadjariensis*) has been determined by the Sanger-sequencing (Kojima 2010). In this study, we provide the nearly complete mitogenome data of this species determined by shotgun sequencing, including all protein-coding genes (PCGs), transfer RNA genes (tRNAs), and ribosomal RNA genes (rRNAs). These mitogenome data represent the first nearly complete mitogenome for the genus and the fourth within the family Potamididae.

A specimen of *P. pupiformis* was obtained from Hitsugaura, Rifu, Miyagi, Japan (38°21′04″N, 141°03′17″E) in November 2018. DNA was extracted from muscle tissue using the Qiagen DNeasy kit according to manufacturer’s protocol and sequenced using a Miseq System (Illumina, San Diego, CA) at the National Institute for Environmental Studies, Tsukuba City, Japan. A total of 23,417,236 reads were assembled in NOVOPlasty (Dierckxsens et al. 2017) with the partial COI sequence of the specimen amplified and sequenced with primers LCO1490 and COI-6 (Shimayama et al. 1990; Folmer et al. 1994) as a seed input. An assembled contig (15,779 bp) was identified as the mitogenome sequence and annotated using the MITOS webserver (Donath et al. 2019), but tandem repeat sequences in the non-coding region were missing. The nearly complete mitogenome sequence was deposited in the DNA Data Bank of Japan (DDBJ) under the accession number LC648322. The sequenced specimen was deposited at Atmosphere and Ocean Research Institute, The University of Tokyo (https://www.aori.u-tokyo.ac.jp, contact person: Shigeaki Kojima, kojima@aori.u-tokyo.ac.jp) under the voucher number H018-Ppupi.

The mitogenome sequence of *P. pupiformis* contains 13 PCGs, 22 tRNAs, and two rRNAs (12S and 16S). Of these 37 genes identified, four PCGs (COIII, CytB, ND1, and ND6), two rRNAs, and 15 tRNAs are encoded on the minor strand. The majority of PCGs contain ATG as the start codon (only ND4 contains GTG as the start codon), and TAA as the stop codon (the stop codon for ND2, ND3, and ND4L is TAG). Gene overlaps are observed between two pairs of genes, CytB–ND6 and NAD4–NAD4L, with the overlapped size of 1 and 7 bp, respectively. The lengths of 22 tRNAs range from 63 to 72 bp. The 12S (953 bp) and 16S (1,379 bp) genes are located between tRNA^Leu^ and tRNA^Ser^. The gene order for *P. pupiformis* is the same as the previously reported mitogenomes of potamidid species (Nguyen et al. 2018; Xu et al. 2019).

The phylogenetic position of *P. pupiformis* within the superfamily Cerithioidea was inferred by the maximum-likelihood (ML) method based on the present and previous mitogenome sequences (Figure 1). The mitochondrial amino acid sequences of 13 PCGs from *P. pupiformis* and 15 cerithioid species previously published were included in the ML analysis. Sequences were aligned separately for each gene using MUSCLE (Edgar 2004) in Translator X (Abascal et al. 2010) with default parameters. Ambiguously aligned positions were removed using Gblocks (Castresana 2000) with an option to allow gap positions in the final blocks for a less stringent selection. Appropriate evolutionary models for each gene were selected using the AICc in ModelTest-NG (Darriba et al. 2020). The ML tree reconstruction was performed in RAxML-NG v.1.0.3 (Kozlov et al. 2019) with 1,000 bootstrap replications. As with the previous mitogenomic studies (e.g. Fukumori et al. 2019; Lee et al. 2019; Choi et al. 2021), the ML tree in this study showed that the superfamily Cerithioidea was recovered as a monophyletic group (bootstrap probability = 100%). The family Potamididae (*Cerithidea* species+*Pirenella pupiformis*) was monophyletic with robust support (100%). The Batillariidae, Turritellidae, and Pachychilidae were shown to be a clade albeit with a moderate bootstrap probability (64%), as with the previous mitogenomic studies (e.g. Hartnell College Genomics Group 2019; Yan et al. 2020). The group of Thiaridae + Paludomidae + Potamididae + Pleuroceridae + Semisulcospiridae was monophyletic (100%), as indicated by the previous study based on partial mitochondrial 16S and nuclear 28S rRNA sequences (group 2 in Strong et al. 2011). The gene orders of species belonging to this group were similar (B in Figure 1), but the locations of tRNA^Arg^ and tRNA^Gln^ were different from those of other cerithioids belonging to the Batillariidae, Pachychilidae, and Turritellidae (A).

**Figure 1.**
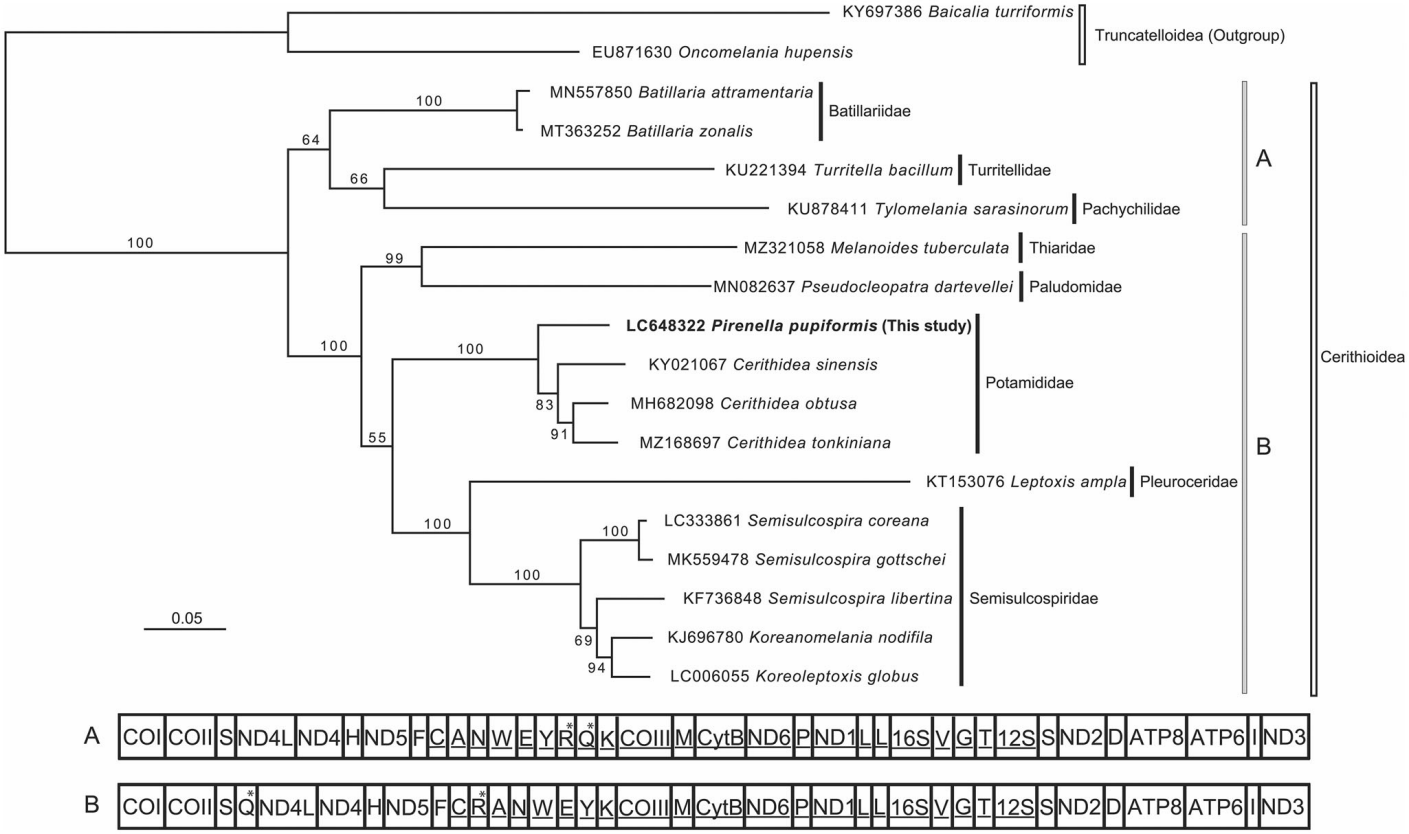
Maximum-likelihood phylogeny of the superfamily Cerithioidea based on mitochondrial amino acid sequences of 13 protein cording genes from *Pirenella pupiformis* (bold, this study; DNA accession number: LC648322) and the 15 cerithioid and two outgroup (Truncatelloidea) species previously published. Accession numbers were shown in the tree. Numbers at node indicate bootstrap probability (BP; 1,000 replicates). Scale bar represents branch length (substitutions/site). Letters (A, B) indicate the group having a different mitochondrial gene order shown under the tree (genes encoded by the minor strand are underlined). Genes whose positions differ between the groups A and B are indicated by asterisks.

## Data Availability

The genome sequence data that support the findings of this study are openly available in the DNA Data Bank of Japan (DDBJ) at http://getentry.ddbj.nig.ac.jp/top-e.html under the accession no. LC648322. The associated BioProject, SRA (DRA), and Bio-Sample numbers are PRJDB12397, DRA012943, and SAMD00409693, respectively.
